# First use of rapid ICT screening for Toxoplasma in pregnant women in Ethiopia: A multicenter study in Bahir Dar, Ethiopia

**DOI:** 10.1371/journal.pntd.0014616

**Published:** 2026-09-30

**Authors:** Yared Mulu Gelaw, Gizachew Worku Dagnew, Mulusew Alemneh Sinishaw, Toyiba Hiyaru, Getu Degu Alene, Jean-Pierre Gangneux, Florence Robert-Gangneux

**Affiliations:** 1 School of Public Health, College of Medicine and Health Sciences, Bahir Dar University, Bahir Dar, Ethiopia; 2 Irset (Institut de Recherche en Santé, Environnement et Travail), UMR_S, Univ Rennes, CHU Rennes, Inserm, EHESP, Rennes, France; 3 College of Medicine and Health Sciences, Assosa University, Assosa, Ethiopia; 4 School of Medical Laboratory Sciences, College of Medicine and Health Sciences, Bahir Dar University, Bahir Dar, Ethiopia; 5 School of Nursing and Midwifery, College of Medicine and Health Sciences, Bahir Dar University, Bahir Dar, Ethiopia; Temple University College of Public Health, UNITED STATES OF AMERICA

## Abstract

**Background:**

*Toxoplasma gondii* primary infection during pregnancy can lead to congenital transmission and cause serious fetal and neonatal complications. Seroprevalence for toxoplasmosis is an indicator of the intensity of transmission and of the risk of infection during pregnancy, yet such data are limited in Ethiopia. This multi-center study utilizing the immunochromatographic test (Toxoplasma ICT IgG-IgM, LD Bio) aimed at determining the seroprevalence of toxoplasmosis among pregnant women and its associated risk factors in Bahir Dar, Ethiopia.

**Methods:**

A cross-sectional study was conducted at two tertiary hospitals. Pregnant women below 20 weeks of gestation were included during the first antenatal care consultation. Participants were selected using systematic random sampling. A blood sample was collected for *Toxoplasma* serology, and socio-demographic and clinical data were collected through a questionnaire. Serology was performed using a rapid immunochromatographic test (ICT Toxo IgG/IgM, LD Bio). The test detects any Toxoplasma antibody (recent or chronic infection), but not separately reported. Data were analysed using SPSS version 23. Individual data were compared between *Toxoplasma*-seropositive and seronegative women to determine risk factors for infection. Bivariable and multivariable binary logistic regression were performed to identify factors associated with seropositivity.

**Results:**

Overall, 1010 women were included in the study at their first antenatal care visit (13.89 ± 4.26 weeks of gestation). Their mean age was 26.8 ± 4.67 years, 85.2% resided in urban areas, and the median monthly income was 5000 Ethiopian Birr. The seroprevalence was 75.8%. Seroprevalence increased with age and parity (p < 0.001), but did not differ according to socio-economic parameters, including educational level, monthly income, occupation, residence, or any hygienic factors. Multivariable analysis showed that age (AOR = 1.09), Parity (AOR = 1.33), eating undercooked meat (AOR = 1.29), and consumption of raw milk (AOR = 1.82) were significantly associated with the seropositivity of T. plasma gondii among pregnant women.

**Conclusion:**

The burden of toxoplasmosis among pregnant women was high in Bahir Dar. Therefore, efforts should be made to increase women’s awareness of risk factors, particularly the habit of consuming raw meat and raw milk. The ease of use of the Toxoplasma ICT test allows serologic screening of pregnant women at antenatal care facilities.

## Author summary

*Toxoplasma gondii* is a zoonotic protozoan parasite that infects both humans and animals. *Toxoplasma* infection during pregnancy causes congenital transmission and serious fetal and neonatal complications. Screening of pregnant women for toxoplasmosis could allow to deliver targeted counselling to seronegative women on how to avoid infection during pregnancy. Additionally, a better knowledge of the seroprevalence could motivate the elaboration of guidelines to reduce toxoplasmosis sequelae.

## Introduction

Toxoplasmosis is an endemic zoonotic disease caused by *Toxoplasma gondii*, a protozoan parasite with a worldwide distribution [1,2]. Cats and other felids are the definitive hosts of the parasite, and humans, as well as virtually all warm-blooded animals, are intermediate hosts, contaminated by ingestion of food or water containing oocysts spread in the environment through cat feces [2]. After primary infection, *Toxoplasma* can disseminate via the bloodstream and gain access to all organs. An immunocompetent host can effectively control this systemic infection, but the parasite cannot be eliminated and persists lifelong as cysts, predominantly located in the muscles, the brain, and the eyes. As a result, the infection can also be acquired through the ingestion of contaminated meat if eaten raw or undercooked [3,4]. In addition, toxoplasmosis may cause congenital infection via transplacental transmission after bloodstream dissemination during primary maternal infection. The infection might vary in severity depending on the stage of pregnancy, possibly resulting in spontaneous abortion or fetal death [5,6].

The seroprevalence of toxoplasmosis among pregnant women varies greatly across countries. Overall, 25.9% and 21.9% seroprevalence rates were reported in France and Portugal, respectively [7,8], similar to that reported in Saudi Arabia (32.5%) [9]. In African countries, the prevalence ranges from below 6% in Zambia and Namibia to above 85% [10]. In Ethiopia, the mean *T. gondii* seroprevalence is high (64%) [10], but reported data vary from 18.5% in Bahir Dar to 94% in Mizan Aman in southeastern Ethiopia, suggesting some heterogeneity [11,12].

Most previous studies have been conducted among pregnant women regardless of their gestation stage, and used miscellaneous serological techniques with various performances. This might make it difficult to understand the magnitude of *Toxoplasma* seropositivity in early pregnancy and hinder the identification of seronegative women who remain at risk of primary infection during gestation, the very group that would benefit most from targeted hygienic counselling. This study aimed to fill this gap by conducting a serological screening of women at the first antenatal care (ANC) consultation and before 20 weeks of gestation, i) to better estimate the proportion of pregnant women at risk of primary infection during pregnancy, and thus the risk of congenital infection, and ii) to evaluate the feasibility of regular screening using the Toxoplasma ICT IgG-IgM (LD Bio, France) test, a highly reliable assay previously validated against reference methods (Architect, Vidas), demonstrating with demonstrated high sensitivity (99.3%) and specificity (100%) [13,14], and iii) to determine the risk factors associated to *Toxoplasma* seropositivity

## Methods and materials

### Ethical consideration

Ethical clearance was obtained from Bahir Dar University, College of Medicine and Health Sciences Ethical Review Board (ERB) with an ethical reference number (CMHS/IRB:10596.2.3) and the Ministry of Education National Research Ethics Review Board with an ethical reference number (FMOE/ERB: 17/152/546/23). An official letter of cooperation was written by the research and development directors to the respective hospitals. Written consent was obtained from all participants after a brief explanation about the objectives of the survey and their rights of participation, including telling them the right to reject any of the questions during the interview if discomfort occurred. Anonymity and confidentiality were ensured for the information obtained from study subjects before the interview.

### Study design, area, and period

A facility-based cross-sectional study was conducted among pregnant women in antenatal care (ANC) clinics of Felege-Hiwot and Tibebe Ghion referral teaching Hospitals, from April 2023 to January 2024. The two investigated hospitals are located in Bahir Dar city, the capital city of the Amhara regional state, and 485 km apart from Addis Ababa, the capital city of Ethiopia. Those comprehensive referral hospitals of the region are serving as centers for teaching, training, and clinical service. The ANC clinic of Felege-Hiwot Hospital provides healthcare for more than 30 pregnant women per day, with 69 beds for both prenatal and postnatal services. On average, more than 35 pregnant women attend prenatal care service at Tibebe Ghion Teaching Hospital every day, with 63 beds for their pre- and post-natal services.

### Study population and eligibility criteria

All pregnant women who attended their first ANC consultation during the study period were eligible to be included in the study. Pregnant women who attended ANC services after 20 weeks of gestation, or who were seriously ill, or who were unable to communicate, were excluded from the study. Whereas, women who refused to provide informed consent were recorded as non-respondents.

### Sample size determination and sampling technique

The sample size was determined using a single population proportion formula, based on a prevalence of 67% taken from a study previously conducted in Debre Tabor, Ethiopia [15], with a 95% confidence interval, a 3% margin of error, and a 10% non-response rate. With this assumption, the sample size was computed as follows:

Sample size (n) = n= $\frac{{\left(Z\alpha /\text{2}\right)}^{\text{2}}p(\text{1}-p)}{{\left(d\right)}^{\text{2}}}$= $\frac{\left(\text{1}.\text{9}\text{6}{)}^{\text{2}}\text{0}.\text{6}\text{7}*(\text{1}-\text{0}.\text{6}\text{7}\right)}{{\left(\text{0}.\text{0}\text{3}\right)}^{\text{2}}}$=944; After adding a 10% non-response rate, the final sample size was 1038.

The study participants were selected using a systematic random sampling technique. The estimated monthly client load of pregnant women attending their first ANC visit at the two referral hospitals was approximately 580 (300 at Tibebe Ghion Hospital and 280 at Felege-Hiwot Hospital). The target total sample size was 1,041. To complete data collection within four months, the required monthly sample size was calculated as 1041÷4=260 per month. The sampling interval (k) was then determined by dividing the estimated monthly client load (580) by the required monthly sample size (260), yielding k = 2.23, rounded to 2. Therefore, every second pregnant woman who met the eligibility criteria was selected. The first participant at each hospital was chosen using a lottery method among the first two women on the first day of data collection, and subsequent participants were selected at every second interval thereafter. Although the data collection period was planned to be over four months, it took more than double that due to the civil war in the area.

### Study variables

#### The dependent variable was: *Toxoplasma gondii* infection (positive serologic test).

Independent variables were collected through a questionnaire (S1 File) and included: (i) socio-demographic variables (age, residence, educational status, type of dwelling, occupation, income, religion), (ii) obstetric variables (weeks of gestation, history of abortion, gravidity, parity, (iii) dietary practice and nutritional status (consumption of raw meat, raw goat milk, uncooked vegetables, sources of drinking water, meals taken outside home, (iv) hygienic and other behavioural factors (presence of a cat in the house, contact with cats, contact with raw meat, contact with raw milk, and contact with cat utensils or litter, contact with soil or gardening, handwashing after activities, and (v) maternal co-morbidities and other variables (HIV infection, Hepatitis-B infection, Blood transfusion, taking of immunosuppressive drugs, such as corticosteroids or chemotherapy).

### Data collection tools, procedures, and quality assurance

A structured English version questionnaire was adapted from different works of literature [13,15–22] that have a related context to the current study. Then it was translated into the local language (Amharic) and retranslated back to English to check its consistency. Data were collected through face-to-face interviews using a Kobo Toolbox. Four trained data collectors who have a BSc degree in midwifery, four laboratory technologists, and two supervisors who have experience in data collection participated in data collection.

To ensure content validity and reliability, the questionnaire language clarity and comprehensibility were extensively reviewed by experts, followed by pre-testing among 5% of participants from the study area to check tool consistency, sensitivity, and local understandability. Additional measures included training for data collectors and supervisors, daily data checks, double data entry for 10% of randomly selected records, and regular supervision visits.

### Samples

The blood sample was taken from all selected women at their first ANC contact (before 20 weeks of gestation). Venous blood was collected in a 5 mL tube containing no anticoagulant and labelled using the medical registration number, questionnaire code, time, and date of sampling.

### Serological diagnosis

After centrifugation, the serum was collected and stored at −20°C until analysis. All collected serum specimens were screened for *T. gondii*-specific IgG and IgM antibodies using a rapid diagnostic test (*Toxoplasma* ICT IgG/IgM, LD Bio, Lyon, France). This assay can detect both specific IgG and IgM in a single band. Briefly, 15 µL of serum was deposited in a cassette, and four drops of buffer were added, following the manufacturer’s recommendations. After 20 min, the validity of the test was assessed by the detection of a control band and the presence of anti-Toxoplasma antibody by the detection of a specific band. Technicians were trained before launching the first inclusions. Although the ICT test is designed for point-of-care, blood samples were collected and serum stored at -20°C to allow batch testing at a central laboratory for quality control purposes. In routine clinical practice, the test can be performed immediately at the ANC clinic laboratory

### Data management and analysis

The data collected via the Kobo Toolbox were exported to the Statistical Package for Social Sciences (SPSS) version 23 software for cleaning and analysis. Quantitative data were expressed as mean **±** SD or interquartile range (IQR). Both descriptive and analytical statistical analyses were done. Variable selection was guided by a priori knowledge from the literature, not purely by statistical criteria. Our analysis used a multivariable binary logistic regression model, a standard method for identifying independent predictors while controlling for confounding. All variables with p-value ≤ 0.2 in bivariable analysis were entered into the multivariable binary logistic regression analysis, based on the principle that confounding can alter p-value |0.15| (i.e., p = 0.2 in bivariable analysis may became below 0.05 after adjustment, and p < 0.05 may rise to above 0.05) and prevent model over fitting Variables with a p-value < 0.05 in multivariable analysis were considered as predictor variables for the seroprevalence of *Toxoplasma* infection. The strength of association was presented using the adjusted odds ratio (AOR) with its corresponding 95% confidence interval (CI). Multicollinearity between each explanatory variable was checked by using standard errors and correlation coefficients. Variables with a correlation coefficient |r| > 0.5 were considered to have moderate to high correlation, indicating potential multicollinearity. When two variables were correlated above this threshold, we removed the variable with less plausible clinical or biological importance for the outcome based on the literature. The chi-square assumption was also checked with the contingency tables of the expected and observed frequencies. Model fitness was checked by using the Hosmer-Lemeshow goodness of fit test; p = 0.918, which is > 0.05, and the model was fit.

## Results

### Sociodemographic, obstetric, and reproductive-related characteristics

A total of 1010 pregnant women participated in the survey with a response rate of 97.3%. The remaining 2.7% were excluded due to incomplete data. Duplicate records were identified by matching medical registration numbers; in each case, the first complete record was retained, and the remaining duplicate entry was deleted from the database. The age of the study participants ranged from 18 to 43 years (mean 26.80 ± 4.67). The mean gestational age of the current pregnancy at the first ANC visit was 13.89 ± 4.26 weeks (ranges from 3 to 20 weeks). More than half, 623 (61.68%), of the participants were in the age group of 25–34 years (Table 1). The majority of the participants declared to reside in urban areas (85.15%), to be Christian followers in religion (93.96%), and were married (99.21%). More than half (53.76%) of the study participants had a monthly income below 5000 Ethiopian birr (ETB); the median monthly income was 5000 ETB (IQR 3500–7071 ETB). More than one-fourth, 295 (29.21%), of the participants had no previous history of pregnancy.

**Table 1:** Socio-demographic and obstetric variables of included women at their first ANC according to *Toxoplasma* serostatus in Bahir Dar, Ethiopia, 2023-2024. (n = 1010).

Variables	Category	No of women N (%)	Positive anti-Toxoplasma IgG/IgM N (%)	p-value *
Age in years	18-24	299 (29.6%)	196 (65.55%)	<0.001
	25-34	623 (61.68%)	489 (78.49%)	
	35-43	88 (8.71%)	81 (92.04%)	
Residence	Rural	15014.85%)	110 (73.33%)	0.469
	Urban	860 (85.15%)	656 (76.28%)	
Educational status	No formal education	108 (10.69%)	86 (79.63%)	0.095
	Primary (grade 1-8)	254 (25.15%)	181 (71.26%)	
	Secondary (grade 9-12)	322 (31.88%)	240 (74.53%)	
	College diploma and above	326 (32.28%)	259 (79.45%)	
Occupation	Housewife	322 (31.88%)	236 (73.29%)	0.540
	Merchant	209 (20.70%)	157 (75.13%)	
	Government employee	189 ((18.71%)	151 (79.89%)	
	Farmer	125 (12.38%)	92 (73.6%)	
	Private employee	97 (9.60%)	77 (79.38%)	
	Daily labourer	68 (6.73%)	53 (77.94%)	
Religion	Christian	949 (93.96%)	720 (75.87%)	0.935
	Muslim	61 (6.04%)	46 (75.41%)	
Monthly income	600-3500	277 (27.43%)	201 (72.56%)	0.178
	3501-5000	266 (26.34%)	196 (73.68%	
	5001-8000	286 (28.32%)	225 (78.67%)	
	>8000	181 (17.92%)	144 (79.56%)	
Parity	Nulliparous	344 (34.06%)	221 (64.24%)	<0.001
	1 to 3	610 (60.40%)	495 (81.15%)	
	4 to 7	56 (5.54%)	50 (89.29%)	
History of abortion	Yes	202 (20.00%)	161 (79.70%)	0.152
	No	808 (80.00%)	605 (74.88%)	
Stage of current gestation^§^	3-8 weeks	139 (13.76%)	107 (76.98%)	0.813
	9-12 weeks	243 (24.06%)	184 (75.72%)	
	13-16 weeks	295 (29.21%)	238 (80.68%)	
	17-20 weeks	333 (32.97%)	247 (74.17%)	

* Chi-square test.

§ weeks of amenorrhea.

Overall, 75.8% of women (95%CI: 72.6-77.8) were positive for anti-*Toxoplasma* IgG/IgM. We have also calculated the adjusted seroprevalence using the Rogan-Galden estimator. Assuming sensitivity 99.3% and specificity 100% [13,14,16], the adjusted seroprevalence became 76.33%, and the 95%CI of the apparent and adjusted prevalence revealed no significant difference (no adjustment needed given near-perfect specificity). Which was computed as follows;


$$\begin{array}{c} \text{True prevalence}=\frac{(Apparent\;prevalence+Specificity)-\text{1}}{(Sensitivity\;+\;Specificity)-\text{1}}=\frac{\text{0}.\text{7}\text{5}\text{8}+\text{1}-\text{1}}{\text{0}.\text{9}\text{9}\text{3}+\text{1}-\text{1}}=\frac{\text{0}.\text{7}\text{5}\text{8}}{\text{0}.\text{9}\text{9}\text{3}}=\text{76}.\text{33}\% \end{array}$$

The seroprevalence was higher among participants aged from 35 to 45 years, compared to 18–24 years (92.04% vs 65.55%, respectively; Chi-square test, p < 0.001). Grand multi-para women had a higher *T.gondii* seroprevalence compared to the nulli-para women (89.29% vs 64.24%, respectively; p < 0.001) (Table 1). However, it did not differ significantly by women’s educational status, area of residence, income level, occupation, religion, or week of gestation (Table 1).

### Hygiene habits and other behaviour-related variables of the study participants

Among participants, 424 (42%) had a habit of eating raw/undercooked meat. Also, 682 (67.5%) and 665 (66%) had a habit of eating raw vegetables and cured meat, respectively. Besides, 276 (27%) women had a habit of feeding outside the home. Around 16% of them had a cat in their home or a history of contact with cats. More than two-thirds (70%) had exposure to soil. The vast majority (91%) were used to washing hands after the manipulation of raw meat (Table 2).

**Table 2:** Nutrition, hygiene, and other behavioral-related variables of the study participants, according to Toxoplasma serological status in Bahir Dar, Ethiopia, 2023-2024 (n = 1010).

Variables	Category	No of women N (%)	Positive anti-Toxoplasma IgG/IgM N (%)	p-value*
Consumption of raw/undercooked meat	Yes	424 (41.98%)	336 (79.24%)	0.032
	No	586 (58.02%)	430 (73.38%)	
Consumption of raw/unpasteurized milk	Yes	422 (41.78%)	351 (83.18%)	<0.001
	No	588 (58.22%)	415 (70.58%)	
Consumption of raw vegetables	Yes	682 (67.52%)	518 (75.95%)	0.905
	No	328 (32.48%)	248(75.61%)	
Eating cured meat	Yes	665 (65.84%)	515 (77.44%)	0.099
	No	345 (34.16%)	251 (72.75%)	
Ways of cooking meat	Boiled	620 (61.39%)	490 (79.03%)	0.009
	Grilled/roasted	98 (9.70%)	72 (73.47%)	
	Oven cooked	292 (28.91%)	204 (69.86%)	
Meals taken outside the home	Never	734 (72.67%)	562 (76.57%)	0.361
	< 1 per week	272 (26.93%)	202 (74.26%)	
	1-3 per week	4 (0.39%)	2 (50.40%)	
Sources of drinking water	Improved	972 (96.24%)	735 (75.62%)	0.400
	Unimproved	38 (3.76%)	31 (81.58%)	
House floor type	Cement	393 (38.91%)	307 (78.12%)	0.178
	Soil	617 (61.09%)	459 (74.39%)	
Cat present at home	Yes	171 (16.93%)	135 (78.95%)	0.298
	No	839 (83.07%)	631 (75.21%)	
Contact with cats	Yes	169 (16.73%)	137 (81.07%)	0.082
	No	841 (83.27%)	629 (74.79%)	
Manipulation of raw meat	Yes	907 (89.80%)	689 (75.96%)	0.786
	No	103 (10.20%)	77 (74.76%)	
Precautions while handling cat feces	Yes	361 (35.74%)	263 (72.85%)	0.098
	No	649 (64.26%)	503 (77.50%)	
Exposure to soil	Yes	710 (70.30%)	550 (77.46%)	0.021
	No	300 (29.70%)	216 (72.00%)	
Hand-washing after handling raw meat	Yes	923 (91.39%)	696 (75.41%)	0.292
	No	87 (8.61%)	70 (80.46%)	
Hand-washing after contact with soil or animals	Yes	845 (83.66%)	645 (76.33%)	0.411
	No	165 (16.34%)	121 (73.33%)	

* Chi-square test.

The seroprevalence of *T. gondii* infection also varied according to the participants’ nutrition-related and hygiene-related variables. Participants who had a habit of eating raw meat had a significantly higher seroprevalence compared to their counterparts (79.24% vs. 73.38%, respectively; p = 0.032). Similarly, the seroprevalence of *T.gondii* infection was higher among women consuming raw milk (83.18% vs. 70.58%, respectively; p < 0.001). A disparity in seroprevalence of *T.gondii* was also observed according to ways of cooking meat (p < 0.01). Participants who had a history of contact with cats had relatively higher *T. gondii* seroprevalence than those who didn’t have (81.07% vs 74.79%, respectively; p = 0.082). On the other hand, participants who took precautions while handling cat feces had a relatively lower risk of acquiring toxoplasmosis infection compared to those who didn’t take precautions (72.85% vs 77.50%, respectively; P = 0.098). Finally, toxoplasmosis seroprevalence was higher in women who had activities in contact with soil (77.46% vs. 72.00%, respectively; p = 0,021) (Table 2).

### Factors associated with *Toxoplasma* infection

In bivariable binary logistic regression analysis, a total of sixteen variables were eligible for multivariable binary logistic regression with a p-value < 0.20. After checking multicollinearity among these independent variables, three variables (monthly income, gravidity, and number of live children) showed correlation coefficients |r| > 0.5 with other variables (e.g., gravidity with parity, r = 0.82) and were therefore excluded from the multivariable model to avoid multicollinearity. Finally, the following variables were included in the multivariable binary logistic regression analysis: age, educational status, parity, consumption of undercooked meat, consumption of raw milk, eating cured meat, ways of cooking meat, having a history of contact with cats, taking precautions while handling cats’ feces, housing floor type, and exposure to soil. In multivariable logistic regression analysis, age, parity, consumption of undercooked meat, and consumption of raw/goat milk had a statistically significant association with seroprevalence of toxoplasmosis (Table 3).

**Table 3:** Factors associated with positive serology for toxoplasmosis among pregnant women in Bahir Dar, multivariate analysis in Bahir Dar, Ethiopia, 2023-2024 (n = 1010).

Variables	Anti-*Toxoplasma* IgG/IgM		COR	AOR (95%CI)
	Positive	Negative		
**Age in years (18–43)**			1.14 (1.10,1.18)	1.09 (1.04, 1.59)
**Educational status**				
Not attend formal education	86	22	1.01 (0.59,1.74)	0.67 (0.37, 1.21)
Primary (grades 1–8)	181	73	0.64 (0.44,0.94)	0.72 (0.47, 1.09)
Secondary (grades 9–12)	240	82	0.76 (0.52,1.09)	0.93 (0.63, 1.38)
College diploma and above	259	67	1	
**Parity (# of delivery) (0–7)**			1.65 (1.42,1.92)	1.33 (1.11,1.58)
**History Abortion**				
Yes	161	41	1.32 (0.90, 1.92)	1.10 (0.74, 1.64)
No	605	203	1	
**Consumption of undercooked meat**				
Yes	336	88	1.39 (1.03,1.87)	1.29 (1.01,1.77)
No	430	156	1	
**Consumption of raw milk**				
Yes	351	71	2.06 (1.51,2.81)	1.82 (1.32,2.50)
No	415	173	1	
**Eating cured meat**				
Yes	515	150	1.29 (0.95,1.73)	100 (0.72, 1.39)
No	251	94	1	
**Ways of cooking meat**				
Boiled	490	130	1.63 (1.19,2.23)	1.09 (0.72, 1.66)
Grilled/roasted	72	26	1.20 (0.72, 2.00)	1.24 (0.72, 2.14)
Oven cooked	204	88	1	
**Have a history of contact with a cat**				
Yes	137	32	1.44 (0.95, 2.19)	1,23 (0.79, 1.83)
No	629	212	1	
**Take precautions while handling cat feces**				
Yes	263	98	1	
No	503	146	1.28(0.96, 1.73)	1.09 (0.78, 1.54)
**Housing floor type**				
Cement	307	86	1	
Soil	459	158	0.81 (0.60,1.10)	1.02 (0.71, 145)
**Exposure to soil**				
Yes	550	160	1.34 (0.98, 1.82)	1.22 (0.88, 1.70)
No	216	84	1	

COR, crude odds ratio; AOR, adjusted odds ratio.

The odds of *T. gondii* infection were increased with an increase in women’s age; when the participants’ age increased by one year, the odds of being positive for T. gondii infection increased by a factor of 1.09 (AOR = 1.09, 95%CI: 1.04, 1.59). Parity (number of deliveries) had a positive association with the seroprevalence of *T. gondii* infection; when the number of births increased by one, the seroprevalence would increase by 1.33 (AOR = 1.33, 95%CI: 1.11,1.58). The habit of eating undercooked meat and drinking raw milk had higher odds for *T. gondii* infection (AOR = 1.29, 95%CI: 1.01,1.77, and AOR = 1.82, 95% CI: 1.32,2.50, respectively) (Table 3).

## Discussion

This study is the first seroprevalence study conducted in Africa, based on the use of the Toxoplasma ICT IgG-IgM test, which is highly suited for developing countries, as it requires no device. The performances of this assay have been previously evaluated in several studies [13,14,16], with a sensitivity and a specificity of 99.3% and 100%, respectively, including in challenging situations, e.g., low IgG titers or very recent infections [14,16]. Its ease of use made it easy for the lab technicians to handle after a short training session, as observed in our study. However, it cannot differentiate anti- IgG and IgM, and detects equally recent and chronic infections. Of note, various ICT assays have been reported in the literature, with discrepant sensitivities. A recent Cameroonian study comparing an ICT and the Vidas Toxo IgG (BioMérieux) showed 13.3% and 80.4% of positive samples, respectively [17].

Our study enrolled the highest number of pregnant women ever (> 1,000), thus had probably more power than previous studies conducted in Ethiopia, and provided robust results. Our results showed a high seroprevalence (75.8%) of *T. gondii* infection among pregnant women in Bahir Dar, consistent with other Ethiopian data from the same area [23,24], or other Ethiopian regions, such as in Bonga Hospital, southwestern Ethiopia (75.7%) [18], and Arba-Minch, southern Ethiopia (79.3%) [15]. This finding suggests that the parasite transmission is very active among the population. However, this seroprevalence estimate (75.8%) reflects lifetime exposure, not necessarily active infection, making it difficult to determine the risk levels of transmitting the infection to the fetus. Besides, it shows that about 24% of women have a risk of acquiring the infection during pregnancy, with associated fetal morbidity. These women should benefit from counselling to avoid infection during pregnancy. Notably, in this study, the mean gestational age at first ANC visit was 13.9 weeks, which reveals that these women are at high risk of acquiring a new infection before giving birth unless they take appropriate preventive measures. Seronegativity varied by age (34.5% among younger aged 18–24 vs.7.9% among those aged 35–43 years), parity (35.8% among multiparous vs. 10.7% among grand multiparous women), dietary habits (e.g., not consuming raw meat or milk). These seronegative women could be specifically targeted for intensive counselling to reduce their risk of primary infection during pregnancy.

This seroprevalence was higher than that reported in neighbouring countries, such as Eritrea (53.6%) [19], Somalia (45.2%) [25], Sudan (22.6%) [26], and Rwanda (12.2%) [27]. Particularly, it was far higher than the seroprevalence reported in Tigray, a neighbouring Ethiopian region (35.6%) [19], and in one study conducted in Bahir Dar (18.5%) [11]. These differences might be due to several factors. First, it can be linked to the sample size, as the study of Awoke et al. [11] included only 384 patients, and to the recruitment of participants (selection mode, socio-demographic characteristics, or cultural habits of a small community). Specific geo-climatic environmental factors, such as desert areas or high tree-lined plateaus, can offer more or less favourable conditions for the survival of *Toxoplasma* oocysts, and thus influence the rate of human infection. Moreover, the sensitivity and specificity of the rapid diagnostic test kit used might also contribute to the observed differences. For example, the latex agglutination test used in the study from Bahir Dar had probably a low sensitivity, and is no longer available on the market in 2025 [11]. Indeed, in other studies conducted in the Amhara region, the seroprevalence was higher [20–22].

The seroprevalence observed in the present study was lower than that reported in other Ethiopian regions, as in Mizan Aman, southwest Ethiopia (94.4%) [12] and Hawassa, southern Ethiopia (81.8%) [24]. The possible explanation for the lower prevalence in the current study, compared to the aforementioned studies, might be due to the difference in the participants’ habit of eating raw meat. In the study done in Hawassa, 59% and 53% of participants had a habit of eating raw meat and unpasteurized milk, respectively [24], while in the current study, 42% of women declared consuming raw meat and unpasteurized milk.

In our study, as in others, the odds of *T. gondii* seropositivity increased with maternal age. Because the ICT test detects any ant-Toxoplasma antibody (reflecting lifetime exposure rather than active infection), this association likely results from cumulative exposure to risk factors over time rather than age-related susceptibility. After adjusting for potential confounders (parity, dietary habits, and cat contact), age remained independently associated with seropositivity. This was in line with previous studies done in Ethiopia [15,18], France [7], Germany [28], Brazil [29,30], and Malaysia [31].

The number of births or parity is an obstetric variable that had a statistically significant association with *T. gondii* infection, probably as a result of higher maternal age that resulted in a cumulative exposure to risk factors over time rather than number of births related susceptibility. Previous pieces of evidence support this finding. In Luanda, Angola, the seroprevalence of *T. gondii* infection increased from 35.6% to 45.9% when the number of births increased from no births to ≥2 births [32]. Similar observations have been described in Ethiopia [18] and Europe [33].

Another important risk factor highlighted in our study was the habit of raw meat consumption, which is commonly practiced in Ethiopia. Similar findings were reported in previous studies done in Ethiopia [15,18,34], and other African countries [27,35–37], as well as in Iran [38], and Europe [39]. In line with our findings, a systematic review and meta-analysis of case-controlled studies identified the consumption of raw meat as a risk factor for acute *T. gondii* infection in humans [40], thus explaining the high seroprevalence in the current study setting. Muscles, together with the brain and retina, are a frequent site for *Toxoplasma* cysts persistence in chronically infected hosts. Consequently, the consumption of raw or undercooked meat is a well-recognized source of infection, as temperatures of at least 65°C are required to inactivate tissue cysts.

Contrasting with some reports [15,19,20,23,24], multivariate analysis did not find any association between seroprevalence of toxoplasmosis and education level, contact with cats, sources of drinking water, hand hygiene after contact with soil, or consumption of raw vegetables, while some factors were statistically significant by univariate analysis.

Importantly, we found that the consumption of unpasteurized milk was a significant risk factor for *Toxoplasma* seropositivity. To our knowledge, very few studies have shown this association through a multivariate analysis [24,37]. Whether women were used to drinking raw cow milk or goat milk is not known, and should be further explored. Indeed, *Toxoplasma* infection in goats is unique, as it can lead to the passage of the parasite in the milk. This passage of *Toxoplasma* tachyzoites in goat milk has been demonstrated by experimental infection of goats [28] and during field studies in lactating animals [29].

Our study has several limitations. The first limitation of our study lies in its cross-sectional design, which precludes the establishment of causal associations between the predictor variables and the outcomes. Second, residual confounding, like biological susceptibility and length of risk exposure not considered in our model. Third, the use of a rapid test detecting both anti-Toxoplasma IgG and IgM cannot distinguish a recent infection from an infection acquired in the past. However, it was very suitable for easy screening of Toxoplasma serostatus, as intended here. A positive ICT serology only indicated the presence of anti-Toxoplasma antibodies. Of course, it could not allow us to determine whether the infection had been acquired before pregnancy or afterwards. The determination of the timing of Toxoplasma infection requires specialized expertise and dedicated laboratory methods, and was beyond the scope of our study. As an observational study, our objective was not to identify and treat women with potentially acute Toxoplasma infection, but rather to assess the feasibility of serological screening under real-world conditions. Indeed, this study was a first attempt to identify women at risk of acquiring toxoplasmosis during pregnancy, to further tailor prevention interventions. We adopted a pragmatic approach that can be readily implemented in low-income countries.

In the setting of further implementation of serologic screening using Toxoplasma ICT, it can be noted that if a recently acquired infection went unnoticed, ultrasound follow-up would likely raise suspicion of congenital toxoplasmosis, as fetal abnormalities are common following infection during the first trimester. We therefore, considered this uncertainty regarding the timing of infection, together with the combined detection of IgG and IgM, to be acceptable.

## Conclusions

Overall, this study performed on a large series confirmed that the seroprevalence of *T. gondii* infection among pregnant women was high in Bahir Dar. The main predictors identified for acquiring *T. gondii* infection were age, parity, consumption of raw meat, and unpasteurized milk. Therefore, efforts should be made in education on proper handling and cooking of meat and milk products. Awareness creation for pregnant women, as well as the community, would be essential to reduce the risk of maternal infection, and thus of congenital transmission. The implementation of a systematic screening using Toxoplasma ICT IgG-IgM could allow to specifically target seronegative women. Initially, our study was designed to retest seronegative women and cord blood at delivery, to check whether infection had occurred during pregnancy, and estimate the incidence of congenital toxoplasmosis. However, due to the strike of the war in the area, most women could not come to the hospital and delivered at home. Only 37 of them benefited from a second serology; they were negative. Further studies are recommended to investigate the impact of maternal *Toxoplasma* infection on fetal outcome. Moreover, future studies in this setting should include confirmatory testing to further validate these findings.

## Supporting information

S1 File English version questionnaire. (DOCX)

## References

[1] Dubey JP. History of the discovery of the life cycle of Toxoplasma gondii. Int J Parasitol. 2009;39(8):877–82. doi: 10.1016/j.ijpara.2009.01.005 19630138

[2] Tenter AM, Heckeroth AR, Weiss LM. Toxoplasma gondii: from animals to humans. Int J Parasitol. 2000;30(12–13):1217–58. doi: 10.1016/s0020-7519(00)00124-7 11113252 PMC3109627

[3] Pereira KS, Franco RMB, Leal DAG. Transmission of toxoplasmosis (Toxoplasma gondii) by foods. Adv Food Nutr Res. 2010;60:1–19. doi: 10.1016/S1043-4526(10)60001-0 20691951

[4] Kijlstra A, Jongert E. Control of the risk of human toxoplasmosis transmitted by meat. Int J Parasitol. 2008;38(12):1359–70. doi: 10.1016/j.ijpara.2008.06.002 18694755

[5] Robert-Gangneux F, Dardé M-L. Epidemiology of and diagnostic strategies for toxoplasmosis. Clin Microbiol Rev. 2012;25(2):264–96. doi: 10.1128/CMR.05013-11 22491772 PMC3346298

[6] Montoya JG, Liesenfeld O. Toxoplasmosis. Lancet Lond Engl. 2004;363:1965–76. doi: 10.1016/S0140-6736(04)16412-X 15194258

[7] Mazzilli S, Tourdjman M, Noël H, Maisa A, Villena I, Le-Ray C, et al. Estimating the national seroprevalence of Toxoplasma gondii infection in pregnant women, France 2021. Epidemiol Infect. 2025;153:e107. doi: 10.1017/S0950268825100502 40916005 PMC12455506

[8] Lobo ML, Patrocinio G, Sevivas T, DE Sousa B, Matos O. Portugal and Angola: similarities and differences in Toxoplasma gondii seroprevalence and risk factors in pregnant women. Epidemiol Infect. 2017;145(1):30–40. doi: 10.1017/S0950268816001904 27608967 PMC9507317

[9] Alghamdi J, Elamin MH, Alhabib S. Prevalence and genotyping ofToxoplasma gondii among Saudi pregnant women in Saudi Arabia. Saudi Pharm J. 2016;24(6):645–51. doi: 10.1016/j.jsps.2015.05.001 27829806 PMC5094437

[10] Mulu Gelaw Y, Worku Dagnew G, Degu Alene G, Gangneux J-P, Robert-Gangneux F. Toxoplasma gondii seroprevalence among pregnant women in Africa: A systematic review and meta-analysis. PLoS Negl Trop Dis. 2024;18(5):e0012198. doi: 10.1371/journal.pntd.0012198 38781272 PMC11152313

[11] Awoke K, Nibret E, Munshea A. Sero-prevalence and associated risk factors of Toxoplasma gondii infection among pregnant women attending antenatal care at Felege Hiwot Referral Hospital, northwest Ethiopia. Asian Pac J Trop Med. 2015;8(7):549–54. doi: 10.1016/j.apjtm.2015.06.014 26276286

[12] Zeleke AJ, Melsew YA. Seroprevalence of Toxoplasma gondii and associated risk factors among HIV-infected women within reproductive age group at Mizan Aman General Hospital, Southwest Ethiopia: a cross sectional study. BMC Res Notes. 2017;10(1):70. doi: 10.1186/s13104-017-2390-6 28126016 PMC5270335

[13] Mahinc C, Flori P, Delaunay E, Guillerme C, Charaoui S, Raberin H, et al. Evaluation of a New Immunochromatography Technology Test (LDBio Diagnostics) To Detect Toxoplasma IgG and IgM: Comparison with the Routine Architect Technique. J Clin Microbiol. 2017;55(12):3395–404. doi: 10.1128/JCM.01106-17 28954897 PMC5703806

[14] Abraham S, Piarroux R, Zhou Y, Tesic V, Abeleda A, Houhou-Fidouh N, et al. Performances of ICT Toxoplasma IgG-IgM test in comparison with Vidas® toxo competition to determine the immune status against Toxoplasma gondii. Eur J Clin Microbiol Infect Dis. 2023;42(11):1327–35. doi: 10.1007/s10096-023-04669-8 37749274

[15] Yohanes T, Zerdo Z, Chufamo N, Abossie A. Seroprevalence and associated factors of Toxoplasma gondii infection among pregnant women attending in antenatal clinic of Arba Minch Hospital, South Ethiopia: Cross sectional study. Transl Biomed. 8. doi: 10.21767/2172-0479.1000105

[16] Rym B-A, Yasmine K, Hedia B, Nesrine I, Olfa S, Rania M, et al. Contribution of the Toxoplasma ICT IgG IgM® test in determining the immune status of pregnant women against toxoplasmosis. J Clin Lab Anal. 2021;35(5):e23749. doi: 10.1002/jcla.23749 33720427 PMC8128306

[17] Yéngué JF, Essomba CMN, Ndzengue GEA, Sonela N, Lobè EE, Tchouaket MCT, et al. Performance of immunochromatographic and immunoenzymatic techniques in the diagnosis of toxoplasmosis in pregnant women in Cameroon: need for harmonization. Pan Afr Med J. 2022;43:195. doi: 10.11604/pamj.2022.43.195.36996 36974314 PMC10038765

[18] Negero J, Yohannes M, Woldemichael K, Tegegne D. Seroprevalence and potential risk factors of T. gondii infection in pregnant women attending antenatal care at Bonga Hospital, Southwestern Ethiopia. Int J Infect Dis. 2017;57:44–9. doi: 10.1016/j.ijid.2017.01.013 28167254

[19] Teweldemedhin M, Gebremichael A, Geberkirstos G, Hadush H, Gebrewahid T, Asgedom SW, et al. Seroprevalence and risk factors of Toxoplasma gondii among pregnant women in Adwa district, northern Ethiopia. BMC Infect Dis. 2019;19(1):327. doi: 10.1186/s12879-019-3936-0 30991956 PMC6469075

[20] Agmas B, Tesfaye R, Koye DN. Seroprevalence of Toxoplasma gondii infection and associated risk factors among pregnant women in Debre Tabor, Northwest Ethiopia. BMC Res Notes. 2015;8:107. doi: 10.1186/s13104-015-1083-2 25879788 PMC4387685

[21] Mulugeta S, Munshea A, Nibret E. Seroprevalence of Anti-Toxoplasma gondii Antibodies and Associated Factors Among Pregnant Women Attending Antenatal Care at Debre Markos Referral Hospital, Northwest Ethiopia. Infect Dis (Auckl). 2020;13:1178633720948872. doi: 10.1177/1178633720948872 32922029 PMC7450295

[22] Endris M, Belyhun Y, Moges F, Adefiris M, Tekeste Z, Mulu A, et al. Seroprevalence and Associated Risk Factors of Toxoplasma gondii in Pregnant Women Attending in Northwest Ethiopia. Iran J Parasitol. 2014;9(3):407–14. 25678926 PMC4316573

[23] Adugna B, Tarekegn ZS, Damtie D, Woldegebreal SN, Raju RP, Maru M, et al. Seroepidemiology of Toxoplasma gondii Among Pregnant Women Attending Antenatal Care in Northwest Ethiopia. Infect Drug Resist. 2021;14:1295–303. doi: 10.2147/IDR.S299106 33883909 PMC8053702

[24] Fenta DA. Seroprevalence of Toxoplasma gondii among pregnant women attending antenatal clinics at Hawassa University comprehensive specialized and Yirgalem General Hospitals, in Southern Ethiopia. BMC Infect Dis. 2019;19(1):1056. doi: 10.1186/s12879-019-4694-8 31842783 PMC6916095

[25] Hassan SA, Tamomh AG, Hassan DA, Ahmed NR. Seroprevalence of anti-Toxoplasma gondii antibodies and associated risk factors in pregnant women in Somalia. Acta Trop. 2023;238:106774. doi: 10.1016/j.actatropica.2022.106774 36417981

[26] M. Mohammed W, Hs A, Ab S, B Mustafa T. Sero-prevalence and associated risk factors of Toxoplasma gondii infection among pregnant women attending Omdurman friendship hospital, Omdurman. Ment Health Addict Res. 2018;3(1). doi: 10.15761/mhar.1000153

[27] Murebwayire E, Njanaake K, Ngabonziza JCS, Jaoko W, Njunwa KJ. Seroprevalence and risk factors of Toxoplasma gondii infection among pregnant women attending antenatal care in Kigali, Rwanda. Tanzania J Hlth Res. 2017;19(1). doi: 10.4314/thrb.v19i1.2

[28] Dubey JP, Verma SK, Ferreira LR, Oliveira S, Cassinelli AB, Ying Y, et al. Detection and survival of Toxoplasma gondii in milk and cheese from experimentally infected goats. J Food Prot. 2014;77(10):1747–53. doi: 10.4315/0362-028X.JFP-14-167 25285492

[29] Gazzonis AL, Zanzani SA, Villa L, Manfredi MT. Toxoplasma gondii in naturally infected goats: Monitoring of specific IgG levels in serum and milk during lactation and parasitic DNA detection in milk. Prev Vet Med. 2019;170:104738. doi: 10.1016/j.prevetmed.2019.104738 31421505

[30] Lozano TdSP, Benitez A, Santos JCd, Navarro IT, Nagata WB, Pinto MdS, et al. Seroprevalence of Toxoplasma gondii and associated risk factors in pregnant women in Araçatuba, São Paulo, Brazil: a multi-level analysis. Microorganisms 2024;12:2183. 10.3390/microorganisms12112183 PMC11596518 39597572

[31] Andiappan H, Nissapatorn V, Sawangjaroen N, Nyunt MH, Lau Y-L, Khaing SL, et al. Comparative study on Toxoplasma infection between Malaysian and Myanmar pregnant women. Parasit Vectors. 2014;7:564. doi: 10.1186/s13071-014-0564-9 25498432 PMC4297455

[32] Vueba AN, Faria CP, Almendra R, Santana P, Sousa M do C. Serological prevalence of toxoplasmosis in pregnant women in Luanda (Angola): Geospatial distribution and its association with socio-demographic and clinical-obstetric determinants. PLoS One. 2020;15(11):e0241908. doi: 10.1371/journal.pone.0241908 33156846 PMC7647088

[33] Olariu TR, Ursoniu S, Hotea I, Dumitrascu V, Anastasiu D, Lupu MA. Seroprevalence and Risk Factors of Toxoplasma gondii Infection in Pregnant Women from Western Romania. Vector Borne Zoonotic Dis Larchmt N. 2020;20(10):763–7. doi: 10.1089/vbz.2019.2599 32589521

[34] Kassie E, Kebede N, Kassa T, Garoma A, Girma M, Asnake Y, et al. Seroprevalence and risk factors for Toxoplasma gondii infection among pregnant women at Debre Markos Referral Hospital, northwest Ethiopia. Trans R Soc Trop Med Hyg. 2024;118(1):61–8. doi: 10.1093/trstmh/trad053 37571984

[35] El-Shqanqery HE, Ibrahim HM, Mohamed AH, El-Sharaawy AA. Seroprevalence of Toxoplasma gondii infection and associated risk factors among asymptomatic pregnant females in Egypt. J Egypt Soc Parasitol. 2017;47:93–100. 30157336

[36] Kamal AM, Ahmed AK, Abdellatif MZM, Tawfik M, Hassan EE. Seropositivity of Toxoplasmosis in Pregnant Women by ELISA at Minia University Hospital, Egypt. Korean J Parasitol. 2015;53:605–10. doi: 10.3347/kjp.2015.53.5.605 PMC4635823 26537040

[37] van der Colf BE, van Zyl GU, Noden BH, Ntirampeba D. Seroprevalence of Toxoplasma gondii infection among pregnant women in Windhoek, Namibia, in 2016. South Afr J Infect Dis. 2020;35:25. doi: 10.4102/sajid.v35i1.25 PMC8378169 34485464

[38] Khademi SZ, Ghaffarifar F, Dalimi A, Davoodian P, Abdoli A. Prevalence and Risk Factors of Toxoplasma gondii Infection among Pregnant Women in Hormozgan Province, South of Iran. Iran J Parasitol. 2019;14(1):167–73. doi: 10.18502/ijpa.v14i1.732 31123482 PMC6511600

[39] Wilking H, Thamm M, Stark K, Aebischer T, Seeber F. Prevalence, incidence estimations, and risk factors of Toxoplasma gondii infection in Germany: a representative, cross-sectional, serological study. Sci Rep. 2016;6:22551. doi: 10.1038/srep22551 26936108 PMC4776094

[40] Belluco S, Simonato G, Mancin M, Pietrobelli M, Ricci A. Toxoplasma gondii infection and food consumption: A systematic review and meta-analysis of case-controlled studies. Crit Rev Food Sci Nutr. 2018;58(18):3085–96. doi: 10.1080/10408398.2017.1352563 29020460

