# Supplementary material for: First use of rapid ICT screening for Toxoplasma in pregnant women in Ethiopia: A multicenter study in Bahir Dar, Ethiopia
Source: PLoS Negl Trop Dis. 2026 Sep 30;20(9):e0014616. doi: 10.1371/journal.pntd.0014616 (PMC13626374; doi:10.1371/journal.pntd.0014616)
Supplement: Supplementary file 1 [file pntd.0014616.s001.docx]

## English version questionnaire

Part I: Socio-demographic questionnaire

|  | Questions | Response category | Remark |
| --- | --- | --- | --- |
| 1. | Residence | 1. Urban 2. Rural |  |
| 2. | Age | _____years |  |
| 3. | Marital status | 1. Single 2. Married 3. Widowed 4. Divorced 5. Others…… |  |
| 4. | Religion | 1. Orthodox Christian 2. Muslim 3. Protestant 4. Catholic 5. Others…… |  |
| 6. | Educational status | 1. Not attending formal education 2. Primary (grade 1-8) 3. Secondary education (grades 9-12) 4. College diploma and above |  |
| 5. | Occupation | 1. Farmer 2. Gov.t employee 3. Private employee 4. Merchant 5. Daily laborer 6. Others…… |  |
| 7. | Monthly income | ____________EBR |  |
| **Part II: Obstetric-related variables** | | | |
| 8. | Gravidae (number of pregnancies) | ______ |  |
| 9. | Parity (number of delivery) | ______ |  |
| 10. | Number of children | ______ |  |
| 11. | History of abortion | 1. Yes 2. No |  |
| 12. | Weak of current pregnancy | ______ |  |
| 13. | Does the current pregnancy want | 1. Yes 2. No |  |
| **Part III: Dietary practice and nutrition** | | | |
| 14. | Consumptions of raw /undercooked meat | 1. Yes 2. No |  |
| 15. | Consumption of raw milk /goat milk | 1. Yes 2. No |  |
| 16. | Consumption of raw vegetables | 1. Yes 2. No |  |
| 17. | Eating cured meat | 1. Yes 2. No |  |
| 18. | Eating raw vegetables | 1. Yes 2. No |  |
| 19. | Drinking unpasteurized milk | 1. Yes 2. No |  |
| 20. | Length of cooking meat | ______minute |  |
| 21. | Feeding outside of the home | 1. Never 2. Sometimes (less than one times per week) 3. Usually (1-3 times per week) 4. Often (more than three/weak) |  |
| 22. | Source of drinking water /water source | 1. Piped water 2. Public taps 3. Standpipes 4. Tube wells 5. Boreholes 6. Protected dug wells 7. Springs 8. Rainwater 9. Unprotected dug 10. Unprotected spring 11. Tanker truck 12. Surface water |  |
|  | **Part IV: Hygienic and other behavioral related factors** | | |
| 23. | House floor type (soil versus cement) | 1. Soil 2. Cement |  |
| 24. | Presence of cats at home | 1. Yes 2. No |  |
| 25. | Contact with cats | 1. Yes 2. No |  |
| 26. | Contact with raw meat | 1. Yes 2. No |  |
| 27. | Contact with Milk | 1. Yes 2. No |  |
| 28. | Do take Precautions during cat face handling | 1. Yes 2. No |  |
| 29. | Exposure to soil | 1. Yes 2. No |  |
| 30. | Hand washing after handling raw meat | 1. Yes 2. No |  |
| 31. | The habit of handwashing after contact with garden soil or domestic animals | 1. Yes 2. No |  |
| **Part V: maternal comorbidities** | | | |
| 32. | HIV status | 1. Negative 2. Positive 3. Not known |  |
| 33. | Hepatitis -B infection | 1. Negative 2. Positive 3. Not known |  |
| 34. | History of blood transfusion | 1. Yes 2. No |  |
| 35. | History of taking immune-suppressive drugs | 1. Yes 2. No |  |

Part VI: screening of Toxoplasma Gondi infection and other laboratory investigation results

| 36. | Maternal Anti-IgG antibody result | 1. Negative 2. Positive |  |
| --- | --- | --- | --- |
| 37. | Maternal Anti-IgM antibody result | 1. Negative 2. Positive |  |
| 38. | Maternal Leucocyte count | ________ |  |
| 39. | Maternal IgG avidity result | _________ |  |
| 40. | Neonatal anti-IgG antibody result | 1. Negative 2. Positive |  |
| 41. | Neonatal anti-IgM antibody result | 1. Negative 2. Positive |  |
